# Pregnancy Complicated by Extrahepatic Portal Vein Occlusion and Portal Vein Thrombosis: A Case Report

**DOI:** 10.7759/cureus.72457

**Published:** 2024-10-26

**Authors:** Shingo Hosomi, Rie Oyama, Chizuko Isurugi, Takanori Sato, Tsukasa Baba

**Affiliations:** 1 Obstetrics and Gynecology, Iwate Medical University, Yahaba-cho, JPN

**Keywords:** extrahepatic portal vein obstruction, heparin injection, portal vein thrombosis, pregnacy complications, premature labour

## Abstract

We report the case of a 38-year-old woman with a history of extrahepatic portal vein obstruction (EHPVO) who became pregnant and developed portal vein thrombosis. She gave birth after intervention by gastroenterology and cardiology. She was referred to our department due to significant leg edema at eight weeks of gestation, and we noticed EHPVO, portal vein thrombosis, and left pulmonary arteriovenous fistula by contrast-enhanced CT. Therefore, subcutaneous heparin injections of 10,000 units/day were started as a preventive anticoagulant therapy. We performed an emergency cesarean section at 36 weeks of gestation. After surgery, the mother was administered a continuous heparin infusion. On the 11th day after surgery, the postoperative progress was good, so heparin was switched to oral warfarin, and the patient was discharged on the same day. During pregnancy, the risk of varicose vein rupture, hyperammonemia, and pulmonary hypertension increases due to an increase in circulating blood volume. This was a case in which careful perinatal management was performed in collaboration with other departments, resulting in a live birth.

## Introduction

Essentially, extrahepatic portal vein obstruction (EHPVO) is a syndrome that leads to portal hypertension due to the obstruction of the extrahepatic portal vein, including the hepatic portal tract. Portal vein hypertension can lead to complications such as liver dysfunction and esophageal varix, therefore, patients need portosystemic shunt surgery. Stewart and Balfour observed portal vein thrombosis in a patient with splenomegaly, ascites, and variceal dilatation in the late 1860s. Kobrich coined the term cavernoma to describe the spongy appearance of portal veins [1,2]. Around 340 to 560 Japanese people per year are affected by this disease and are treated as either outpatients or inpatients. It is slightly more common among males, with a male-to-female ratio of approximately 1:0.6. A definitive diagnosis is most commonly made among people under 20 years of age, followed by those in their forties and fifties [3]. Generally, a hypercoagulable state, intra-abdominal infection/peritonitis, and portal vein anomaly (portal vein stenosis and atresia) are considered important predisposing factors of EHPVO. However, the vast majority of cases are due to primary thrombosis of the portal vein and often with more than one cause. In addition, the risk of infection is increased. The increased blood volume and cardiac output increase portal flow and aggravate portal hypertension during pregnancy. This increases the risk of variceal bleeding in pregnant women, which can compromise the perinatal outcome of the pregnancy. Endoscopic sclerotherapy (EST) or endoscopic variceal ligation (EVL) reduces the risk of variceal bleeding and can improve the pregnancy outcome in these women [4]. We report a pregnant woman whose complicated EHPVO with portal vein thrombosis and antithrombin (AT)-III deficiency was successfully treated in collaboration with the Department of Pediatrics, Gastroenterology, and Cardiology.

## Case presentation

The patient is a 38-year-old primigravida. After vomiting blood, the esophageal varix was discovered, and the cause was diagnosed as extrahepatic portal vein obstruction at six years old. She underwent an extrahepatic venous shunt surgery. Subsequently, the patient had regular checkups at her family doctor's office, but at the age of 16, she developed hyperammonemia, which caused hepatic encephalopathy. Since then, no imaging tests have been performed because no serious complications were observed. 

**Figure 1:**
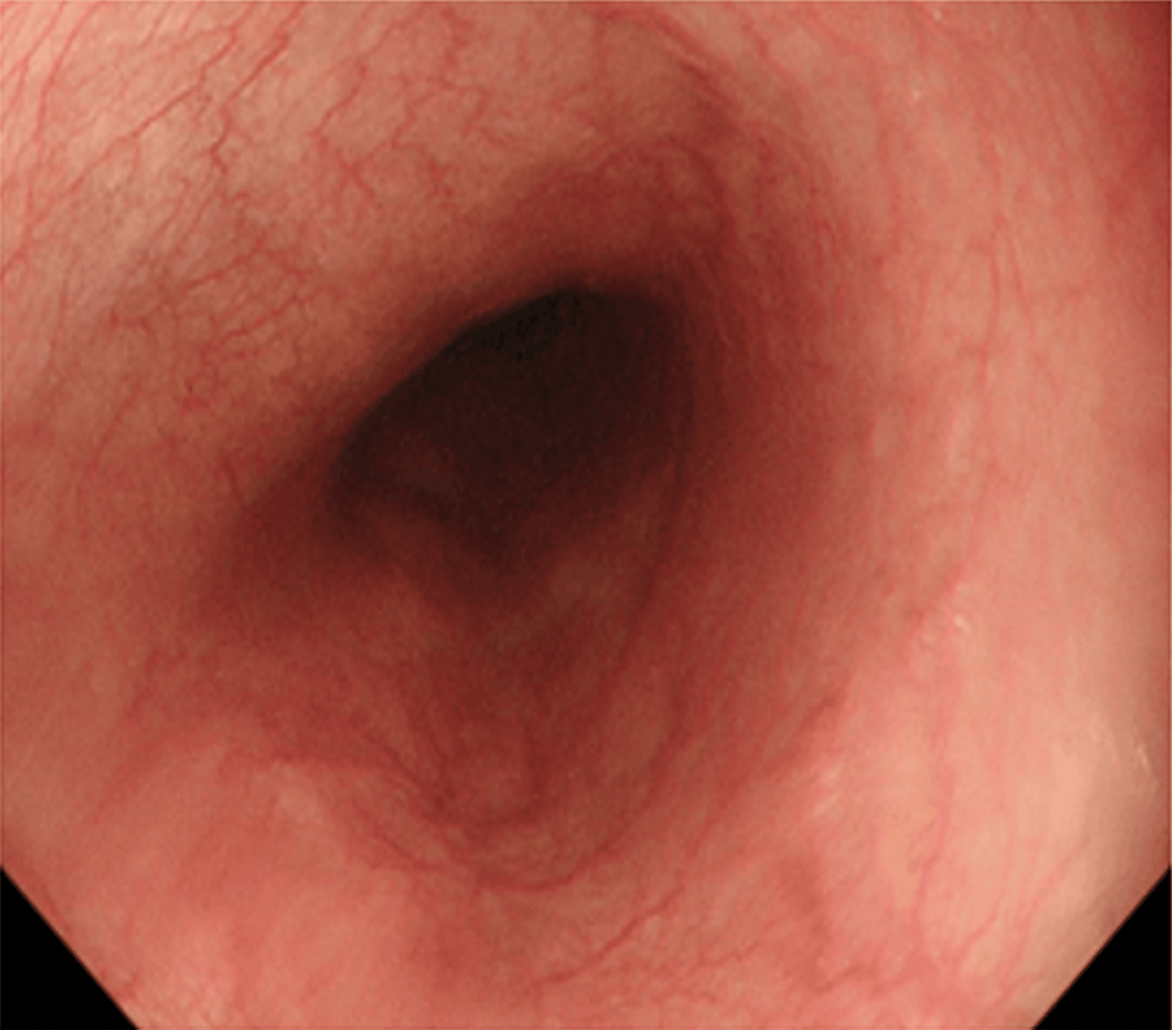
Upper gastrointestinal endoscopy at 13 weeks and four days shows no varicose vein recurrence

**Figure 2:**
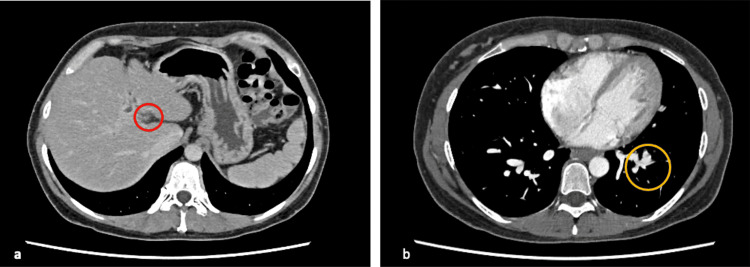
Contrast-enhanced CT performed at 17 weeks and 14 days a: Extrahepatic portal vein occlusion (red circle); b: Left pulmonary venous fistula (yellow circle)

The patient had leg edema since early pregnancy, and spironolactone was initiated to avoid right-sided heart failure. At eight weeks and six days of pregnancy, the patient was referred to our hospital by her previous doctor and family doctor for a thorough evaluation of the EHPVO and pregnancy management. The patient was admitted to the hospital for perinatal management at 10 weeks and one day of gestation. Table 1 shows the blood test results on admission to our hospital. Upper gastrointestinal endoscopy revealed no varicose vein recurrence after 13 weeks and four days (Figure 1). A contrast-enhanced CT was performed at 17 weeks and four days (Figure 2), which revealed an extrahepatic portal vein occlusion and a thrombus in the left pulmonary venous fistula. We suspected AT-III deficiency after noticing AT-III activity at 59% at 18 weeks and two days( Table 1). The AT-III gene testing revealed no genetic abnormalities.

**Table 1:** Blood test results on admission Antithrombin III shows low values. However, liver enzymes were normal.

Tests	Result	Units	Tests	Result	Units
White blood cells	6560	/μL	Total cholesterol	133	mg/dL
Red blood cells	380×10^4^	/μL	High-density lipoprotein cholesterol	45	mg/dL
Hemoglobin	12.7	g/dL	Triglyceride	38	mg/dL
Hematocrit	37.9	%	Amylase	81	U/L
Platelets	15.0×10^4^	/μL	Ammonia	75	μg/dL
Activated partial thromboplastin time ratio	1.25		Protein induced by vitamin K antagonist 2	29	mAU/mL
Prothrombin time	14.1	/sec	Hepatitis B surface antigen	<0.05	IU/mL
Prothrombin time - international normalized ratio	1.17		Hepatitis C virus antibody	0.1	COI
Fibrinogen	293	mg/dL	Protein C activity	52	%
Antithrombin III	59	%	Protein S antigen	46	%
D-dimer	2.4	μg/dL	Potassium	3.4	mEq/L
Total protein	5.9	g/dL	Urea nitrogen	5	mg/dL
Sodium	139	mEq/L	Alanine aminotransferase	23	IU/L
Chlorine	108	mEq/L	γ-glutamyl transpeptidase	29	IU/L
Creatinine	0.59	mg/dL	C-reactive protein	<0.10	mg/dL
Lactate dehydrogenase	193	IU/L	Total bilirubin	0.7	mg/dL

**Figure 3:**
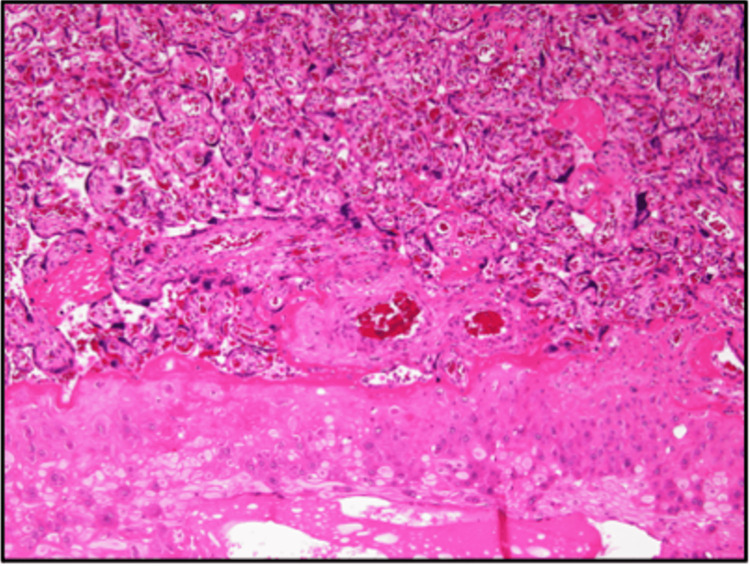
Pathological image of the placenta Hematoxylin and eosin staining revealed no placental hemangioma or ischemic changes in the placental villi.

We decided to start prophylactic anticoagulation with a subcutaneous heparin injection of 10,000 units/day as soon as possible. Echocardiography performed at 21 weeks and one day of gestation revealed no pulmonary hypertension; cardiac function tests were unremarkable. At 36 weeks and one day of pregnancy, fetal heartbeats became non-reassuring fetal status and strong uterine contractions, and an emergency cesarean section was performed on the same day. The operative findings included yellowish amniotic fluid, umbilical cord, and yellowing of the omentum. The uterus, bilateral adnexa, and the pouch of Douglas were normal. No abnormal vessels were visible in the abdominal wall, intestine, or mesentery. The blood loss was 1610 g (including amniotic fluid) during the emergency cesarean delivery. The newborn was female, and her body weight was 2580 g. Hematoxylin and eosin staining the placenta after the emergency cesarean section showed no placental hemangioma or ischemic changes in the placental villi (Figure 3).

Continuous heparin infusion was initiated for the patient on postoperative day one, and 800 mL of red blood cell concentrate was administered on postoperative day three because the hemoglobin level was 7.5 mg/dL. On postoperative day 11, the patient was switched from heparin to warfarin owing to good progression and was discharged from the hospital on the same day. The patient was followed up regularly by the Department of Hepatology at the hospital.

## Discussion

The primary goal of EHPV and portal vein thrombosis treatment is the management of portal vein hypertension (esophageal and gastric varix, splenomegaly, and hypersplenism) and preventing thromboembolism. Pulmonary vein thrombosis was first reported by Eiiot et al. and can be acute, subacute, or delayed in onset [5]. First, Amitrano et al. reported that EHPVO accounts for 80% of pediatric pulmonary hypertension cases [6]. In cases with delayed onset, symptoms are often not observed because of the development of collateral vessels. Second, the etiology of portal vein thrombosis can be idiopathic or secondary to underlying diseases [7,8]. Thrombus formation begins in peripheral branches. The clinical findings of portal vein thrombosis differ between patients with acute and chronic thrombosis [9].

In this case, in the early stages of this pregnancy, the patient had significant bilateral leg edema, and deep vein thrombosis was suspected to be one of the causes; therefore, she was admitted to the hospital for examination. Hoekstra et al. reviewed 45 cases of pregnancy complicated by portal vein thrombosis and reported that the cause was thrombus formation in 27 cases (60%), local factors in 10 cases (22%), and idiopathic factors in eight cases (18%). Anticoagulation therapy was administered in 38 patients (66%), of whom 21 (36%) used low-molecular-weight heparin [10]. 

Our patient underwent extrahepatic venous shunt surgery at the age of eight years and was administered diuretics to prevent right ventricular overload post-surgery. In addition to portal vein thrombosis related to shunt formation and oral diuretics, AT-III deficiency, protein C, and protein S were observed. These abnormalities have been speculated to be caused by chronic liver dysfunction [11]. Pregnancy and AT-III deficiency are known risk factors for venous thromboembolism [12].

The character of normal pregnancy is that coagulation activity is 1.5 to 2 times higher in the late stages of pregnancy than in non-pregnant women [13]. Coagulation system fibrinolytic activity significantly increases after 28 weeks of pregnancy [14]. After consulting with multiple professionals about the pathophysiology of EHPVO, we decided that it was necessary to perform an imaging examination to determine the status of EHPVO and the presence or absence of thromboembolism. After explaining the risks of contrast-enhanced CT to the patient and her family and receiving consent, the scan was performed to seek thrombosis. The imaging diagnosis was portal vein thrombosis. To prevent increased thrombosis and the organization of the portal vein thrombus, we selected the intravenous administration of heparin, which is safe for use during pregnancy [15]. The thromboembolism passes through the pulmonary vein fistula. It causes secondary pulmonary arterial hypertension, pulmonary vascular endothelial damage, and pulmonary vasospasm, potentially leading to hypoxia due to ventilatory failure and decreased blood flow [16].

We considered increased coagulation and fibrinolytic activity, physiological changes during pregnancy, pulmonary thromboembolism due to increased thrombus size, and aggregate formation due to thrombus surface detachment. Heparin administration therapy can be said to be an inevitable treatment. Furthermore, this case report highlights the importance of early evaluation and prevention of pulmonary thromboembolism. Pregnant women with complicated EHPVO and portal vein thrombosis should receive careful management to prevent pulmonary thromboembolism from the early stages of pregnancy.

## Conclusions

Extrahepatic portal vein obstruction with portal vein thrombosis is a syndrome that possibly leads to portal hypertension and thromboembolism. Portal hypertension during pregnancy can cause complications such as liver dysfunction, esophageal varix, aggravation, and organization of venous thrombosis due to AT-III deficiency, and pulmonary hypertension. Therefore, it is necessary to establish a perinatal management network involving multiple professionals within the hospital, carefully observing the patient, and thoroughly explaining the need for examinations before conducting contrast CT scans, regular blood tests, cardiac function tests, and other tests to confirm the presence or absence of thromboembolism. If thrombosis is found, it is necessary to consider safe anticoagulant therapy for pregnant women to avoid hypoxemia due to secondary pulmonary vascular disorders. Finally, pregnant women with EHPVO and portal vein thrombosis need careful management to prevent pulmonary embolism from early pregnancy and continued preconception care throughout childhood and adulthood.
